# Supplementary material for: Repetitive mild head trauma induces activity mediated lifelong brain deficits in a novel Drosophila model
Source: Sci Rep. 2021 May 6;11:9738. doi: 10.1038/s41598-021-89121-7 (PMC8102574; doi:10.1038/s41598-021-89121-7)
Supplement: Supplementary file 1 — Supplementary Figures. [file 41598_2021_89121_MOESM1_ESM.pdf]

Repetitive Mild Head Trauma Induces Activity-Mediated Lifelong Brain Deficits in a Novel Drosophila Model

Joseph A. Behnke, Changtian Ye, Aayush Setty, Kenneth H. Moberg, and James Q. Zheng

Supplemental Figures S1-S8 (Behnke et al.)

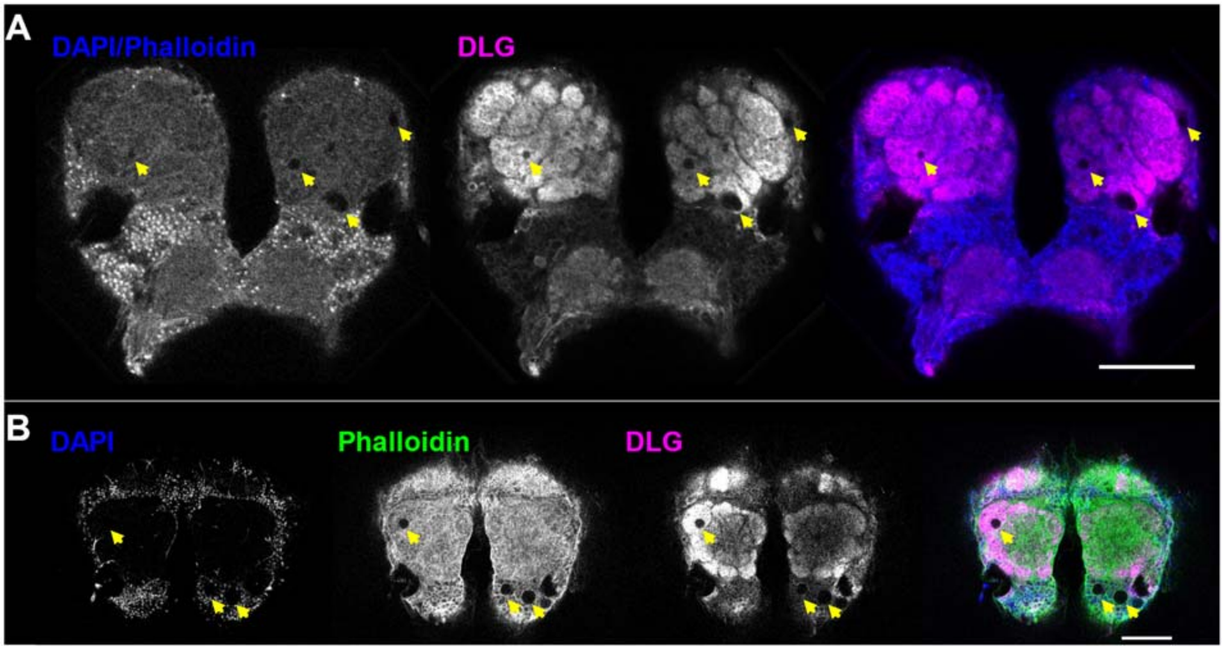

Supplemental Figure S1

Detecting Neurodegeneration in Drosophila Whole-Brain Mounts (**a**) Two-Photon Microscopy of whole-brain mounts stained with DAPI and phalloidin (blue) to detect brain parenchyma and discs large 1 (DLG, magenta) to detect post-synaptic neuropil. (**b**) Confocal Microscopy of whole-brain mounts stained with DAPI (blue) to detect nuclei, phalloidin (green) to detect brain parenchyma, and discs large 1 (DLG, magenta) to detect post-synaptic neuropil. Yellow arrows designate vacuoles (absence of signal), scale bar = 50μm.

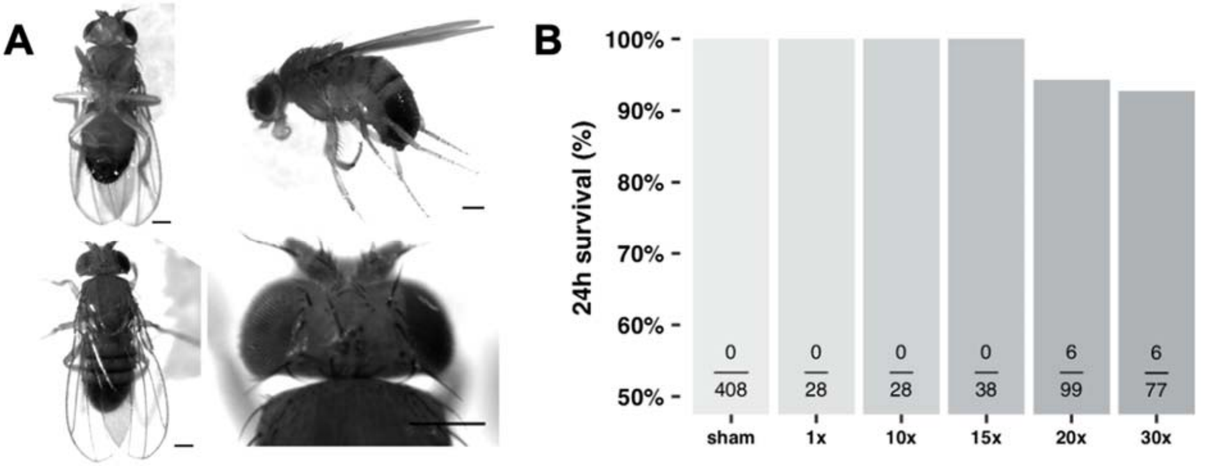

Supplemental Figure S2

Gross Morphology and Acute Survival Following Repetitive Head Impacts (a) Representative whole-body micrograph of an injured *Oregon R* male fly showing no signs of gross morphological damage to the head or body following repetitive head impact exposure. Scale bar= 100 µm**. (b)** Barplot of acute survival (24h) following varying number of iterative successive head impacts delivered 10s apart. Black text indicates (# dead/# at risk).

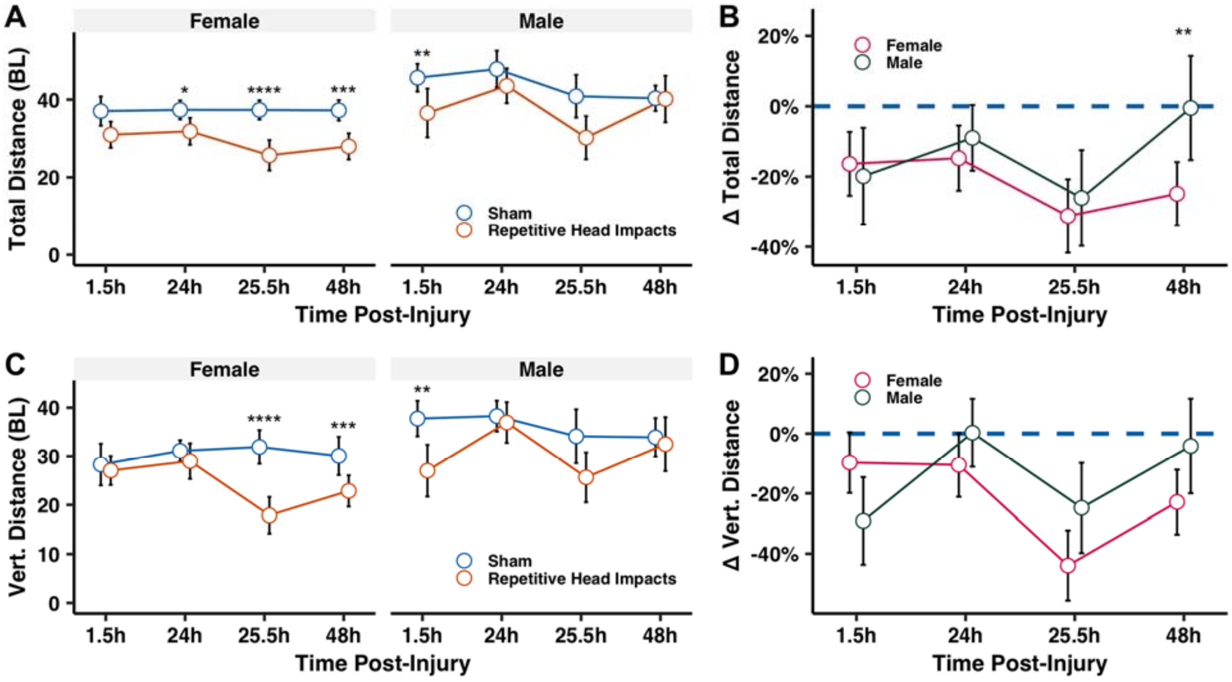

Supplemental Figure S3

Acute recovery of climbing deficits following minimally lethal repetitive head impacts is sexually dimorphic. Repetitive head impacts elicit acute climbing deficits in both male and female, seen as a reduction in **(a)** total climbing distance and **(c)** total vertical distance traversed during the startle-induced climbing assay. **(b&d)** Injured female flies show progressive relative behavioral deficits that worsen after the second impact session, while injured male flies show active acute recovery 24h after each session of impact. Plotted values are median **(a&c)** raw or **(b&d)** relative (to respective sex) values with 95% confidence intervals as error bars. Mann–Whitney *U* test between **(a&c)** injured and non-injured groups, with Holm correction and **(b&d)** injured female and male performance relative to non-injured, with Bonferroni correction. *p<0.05, **p<0.01, ***p<0.001, ****p<0.0001, n=25-35 flies per sex/time/injury group.

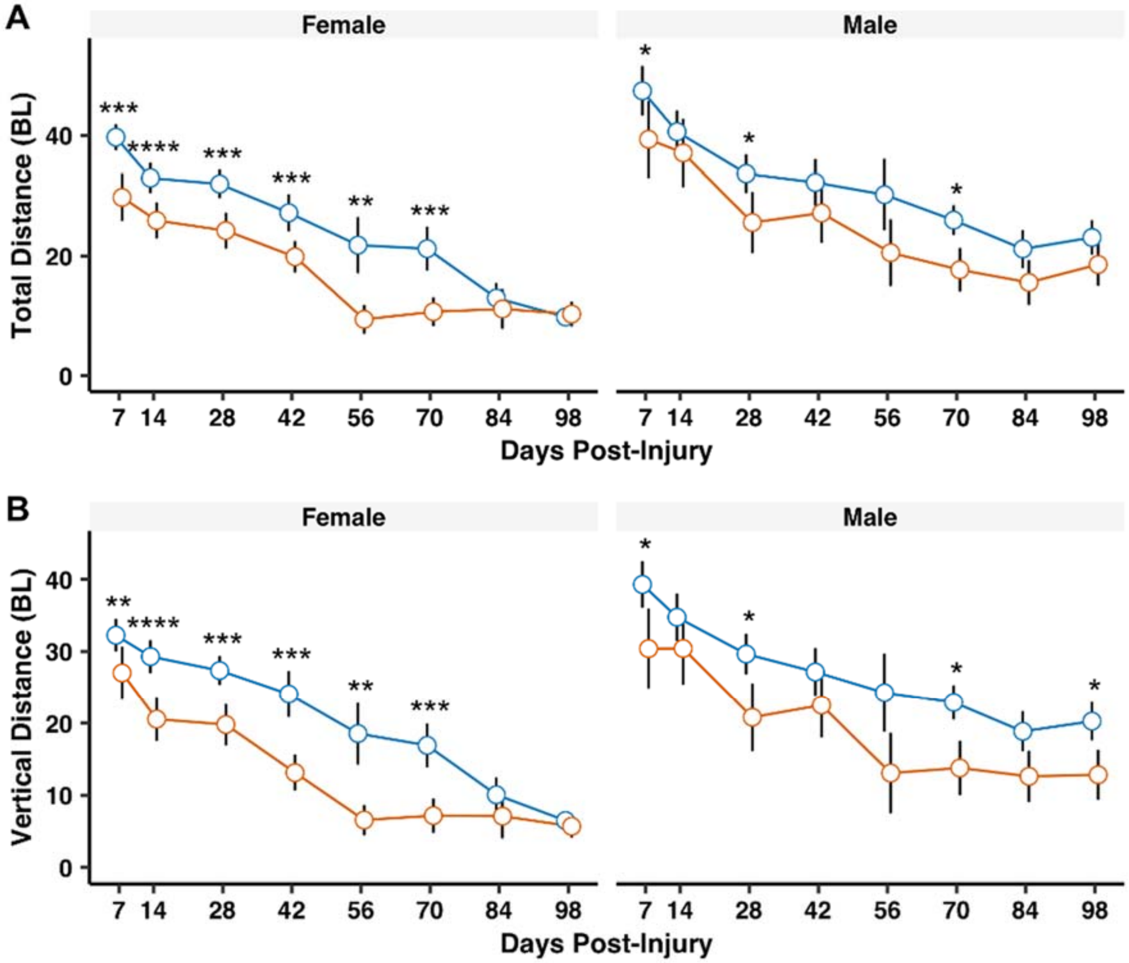

Supplemental Figure S4

Repetitive head impacts result in long-term behavioral climbing deficits. Repetitive head impacts elicit chronic climbing deficits that are more pronounced in female flies, seen as a reduction in **(a)** total climbing distance and **(b)** total vertical climbing distance traversed during the climbing assay. Plotted values are median values with 95% confidence intervals as error bars. Mann–Whitney *U* test between injured and non-injured groups, with Holm correction: *$p<0.05$, **$p<0.01$, ***$p<0.001$, ****$p<0.0001$, $n \geq 22$ flies per sex/time/injury group except day 56 $n \geq 11$ flies.

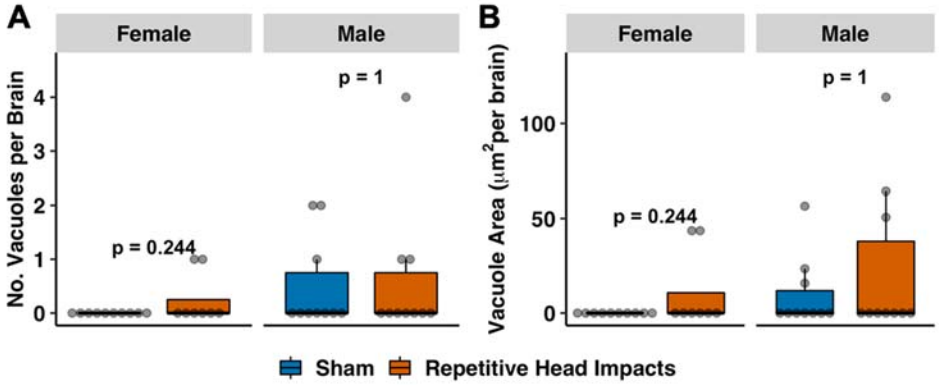

Supplemental Figure S5

Repetitive head impacts result in no acute neurodegeneration. Repetitive head impacts elicit no acute (1.5h post-injury) neurodegeneration, neither seen as an **(a)** increased number of vacuoles and **(b)** vacuole area per brain. Boxplots contain individually plotted values with whiskers corresponding to the maximum 1.5 interquartile range. Within sex differences between sham and repetitive head impact conditions were analyzed with the Mann–Whitney *U* test with Bonferroni correction.

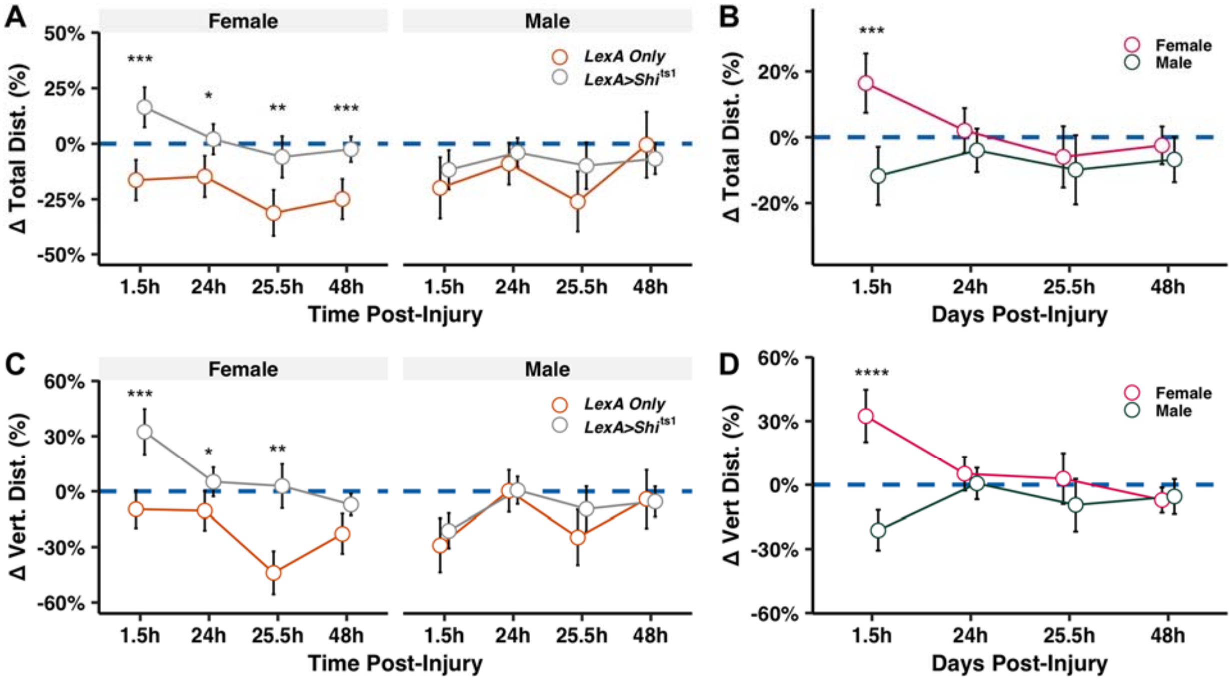

Supplemental Figure S6

Suppressing acute injury-induced neuronal activity following repetitive head impacts preferentially benefits females. Blocking activity protects against acute climbing deficits in female flies, specifically **(a&b)** relative total distance and **(c&d)** relative vertical distance traversed. Plotted values are relative median values (compared to respective genotype sham) with 95% confidence interval error bars. Differences in relative climbing behavior were analyzed using the Mann–Whitney *U* test with Holm correction, between injured *LexA Only* and *Shi*^{ts1}-containing flies. *p<0.05, **p<0.01, ***p<0.001.

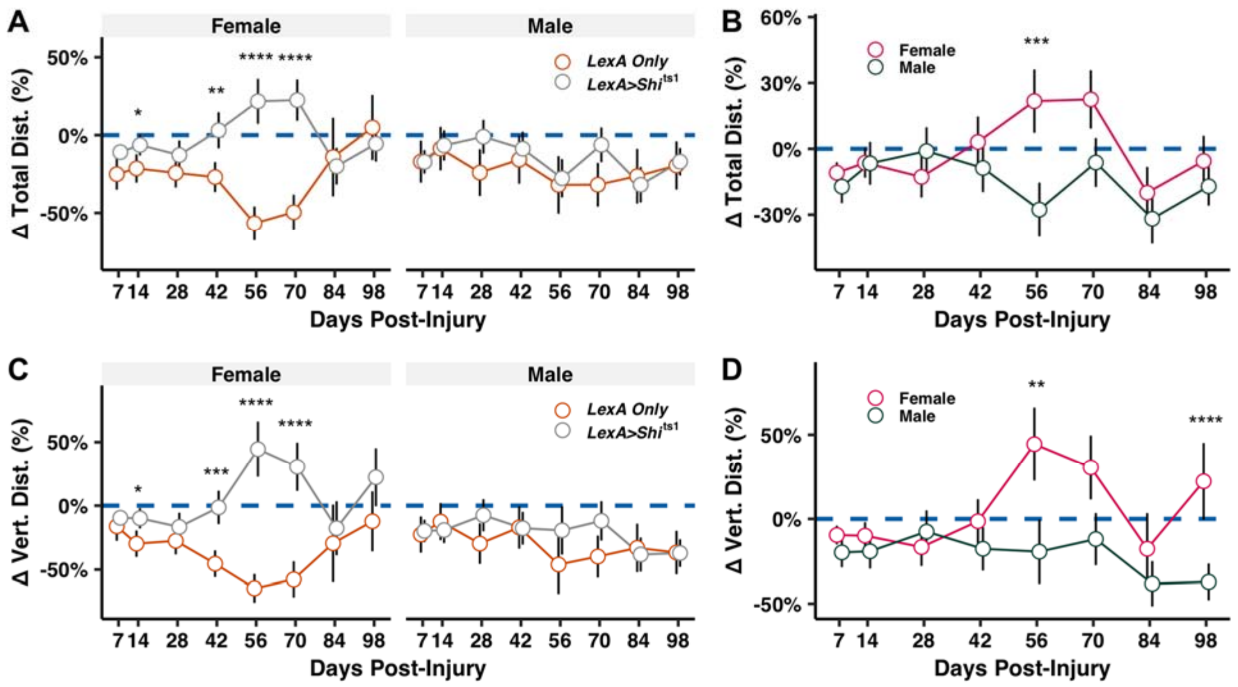

Supplemental Figure S7

Suppressing acute injury-induced neuronal activity following repetitive head impacts preferentially benefits females. Blocking activity protects against chronic climbing deficits in female flies, specifically **(a&b)** relative total distance and **(c&d)** relative vertical distance traversed. Plotted values are relative median values (compared to respective genotype sham) with 95% confidence interval error bars. Differences in relative climbing behavior were analyzed using the Mann–Whitney *U* test with Holm correction, between injured *LexA Only* and *Shi*^{ts1}-containing flies. *p<0.05, **p<0.01, ***p<0.001.

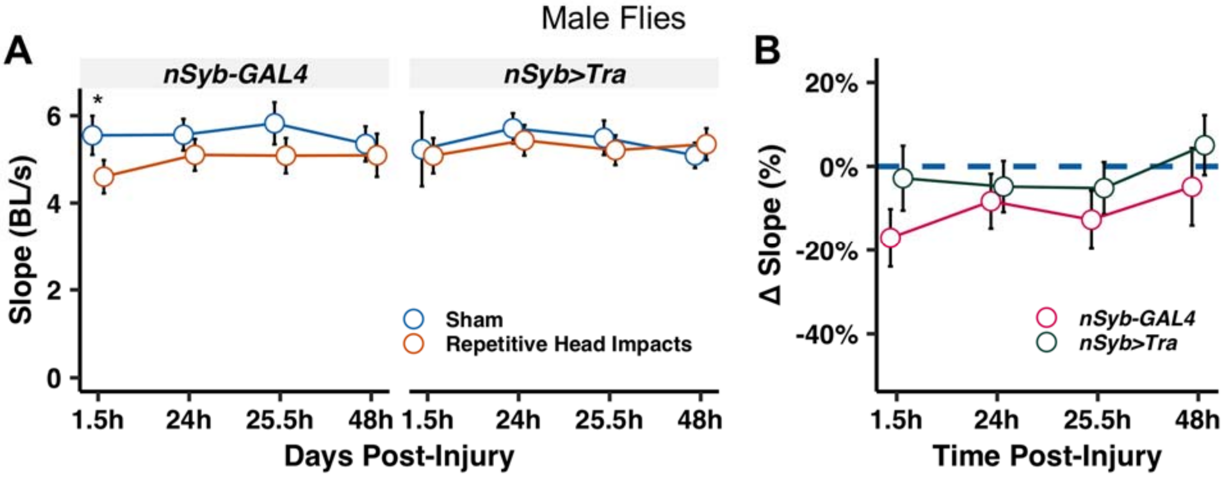

Supplemental Figure S8

Climbing effect of pan-neuronal expression of the female-specific splicing regulator *transformer* in male flies following repetitive head impacts. Male flies with (*nSyb>Tra*) and without (*nSyb-GAL4*) pan-neuronal *transformer* expression were subjected two sessions of repetitive head impacts as described in Fig. 2. Their startle-induced climbing behaviors were assessed at various points before and after the repetitive head impacts. **(a)** Plotted climbing slope of sham and injured male flies with (*nSyb-Tra*) and without *transformer* (*nSyb-GAL4*). **(b)** Plotted relative climbing slope of injured male flies with (*nSyb>Tra*) and without *transformer* (*nSyb-GAL4 Only*). (Δ slope= (Injured Slope-Median Sham Slope)/Median Sham Slope). Plotted values are median values with 95% confidence interval error bars. Differences in relative climbing behavior were analyzed using the Mann–Whitney *U* test with Holm correction, between **(a)** sham and injured male flies or **(b)** injured *GAL4 Only* and *Transformer*-containing flies. $^*p<0.05$.
