# Supplementary material for: Repetitive mild head trauma induces activity mediated lifelong brain deficits in a novel Drosophila model
Source: Sci Rep. 2021 May 6;11:9738. doi: 10.1038/s41598-021-89121-7 (PMC8102574; doi:10.1038/s41598-021-89121-7)
Supplement: Supplementary file 6 — Supplementary Information. [file 41598_2021_89121_MOESM6_ESM.docx]

**Supplementary Material:**

**Supplemental figures:** S1 – S8.

**Supplemental video:** Video S1-S4

**Supplemental video S1.mp4:**

**Demonstration of headfirst impacts using our novel *Drosophila* model** Multiple unrestrained awake flies contained within a plastic injury vial are accelerated upward until the vial reaches the top of the apparatus, after which forward momentum of the flies carries them further upward until they impact the upper surface of the vial where they sustain headfirst impacts

**Supplemental video.mp4:**

**Head impacts produce immediate concussive-like behavior** Flies sustain acute signs of neurological injury immediately following head impacts, such as temporary loss of consciousness and uncoordinated behaviors that become more prevalent with increasing number of repetitive head impacts.

**Supplemental video S3.mp4:**

**Automated tracking of fly climbing using idtracker.ai** Individual fly climbing behavior within the startle-induced negative geotaxis assay is analyzed using an automated tracking algorithm idtracker.ai.

**Supplemental video S4.mp4:**

**Novel approach to measure frank neurodegeneration in *Drosophila* whole-brain mounts** Two-photon microscopy stack of whole-brain mount stained for DAPI (nuclei) and phalloidin (actin for brain parenchyma). Regions devoid of DAPI/phalloidin that are shaded in green correspond to physiologically normal holes while red shaded regions correspond to pathological vacuoles.
